# Synthesis and Characterization of a Heterometallic Extended Architecture Based on a Manganese(II)-Substituted Sandwich-Type Polyoxotungstate

**DOI:** 10.3390/ma11010155

**Published:** 2018-01-17

**Authors:** Masooma Ibrahim, Eufemio Moreno-Pineda, Thomas Bergfeldt, Christopher E. Anson, Annie K. Powell

**Affiliations:** 1 Institute of Nanotechnology (INT), Karlsruhe Institute of Technology (KIT), Postfach 3640, 76021 Karlsruhe, Germany; eufemio.pineda@kit.edu; 2 Institute for Applied Materials (IAM-AWP), Karlsruhe Institute of Technology (KIT), Postfach 3640, 76021 Karlsruhe, Germany; thomas.bergfeldt@kit.edu; 3 Institute of Inorganic Chemistry, Karlsruhe Institute of Technology (KIT), Engesserstrasse 15, 76131 Karlsruhe, Germany; christopher.anson@kit.edu

**Keywords:** heterometallic, 3d-4f polyoxometalate, molecular magnetism, luminescence, pomif, extended frameworks, trilacunary, wells-dawson polyanion, sandwich-type

## Abstract

The reaction of [*α*-P_2_W_15_O_56_]^12−^ with Mn^II^ and Dy^III^ in an aqueous basic solution led to the isolation of an all inorganic heterometallic aggregate Na_10_(OH_2_)_42_[{Dy(H_2_O)_6_}_2_Mn_4_P_4_W_30_O_112_(H_2_O)_2_]·17H_2_O (**Dy_2_Mn_4_-P_2_W_15_**). Single-crystal X-ray diffraction revealed that **Dy_2_Mn_4_-P_2_W_15_** crystallizes in the triclinic system with space group P$\bar{1}$, and consists of a tetranuclear manganese(II)-substituted sandwich-type phosphotungstate [Mn_4_(H_2_O)_2_(P_2_W_15_O_56_)_2_]^16−^ (**Mn_4_-P_2_W_15_**), Na, and Dy^III^ cations. Compound **Dy_2_Mn_4_-P_2_W_15_** exhibits a 1D ladder-like chain structure based on sandwich-type segments and dysprosium cations as linkers, which are further connected into a three-dimensional open framework by sodium cations. The title compound was structurally and compositionally characterized in solid state by single-crystal XRD, powder X-ray diffraction (PXRD), Fourier-transform infrared spectroscopy (FTIR), thermogravimetric (TGA), and elemental analyses. Further, the absorption and emission electronic spectra in aqueous solutions of **Dy_2_Mn_4_-P_2_W_15_** and **Mn_4_-P_2_W_15_** were studied. Also, magnetic properties were studied and compared with the magnetic behavior of [Mn_4_(H_2_O)_2_(P_2_W_15_O_56_)_2_]^16−^.

## 1. Introduction

Polyoxometalates (POMs) are discrete early transition metal-oxide cluster anions whose physicochemical properties can be tuned by the incorporation of additional transition metal centers [1,2]. A large number of transition metal containing POMs have been synthesized and characterized with enhanced properties, for instance, robustness and stability emerging from the POM moiety and the predictable functional properties, depending on their electronic nature, originating from the redox metal centers. To date, transition metal (3d), lanthanide (4f), and mixed 3d-4f containing POM hybrids have been studied, which can be broadly classified as follows [3,4,5,6,7,8,9].

(a) Three-dimensional (3d)-POMs [10,11,12,13,14]
(b) 4f-POMs [4,15]
(c) 3d-4f-POM [16,17]
(d) 4f-POM with 3d linkers [7,8]
(e) 3d-POM with 4f linkers [7,8]

There are many reports on 3d-POMs, where the number of incorporated 3d metal centers within POM system ranges from 1 to 40 [18]. When compared to 3d-POMs there are fewer publications on 4f-POMs but still a significant number of structures have been characterized and studied. Usually this class of POMs results in large assemblies for example [As_12_Ce_16_W_148_O_524_(H_2_O)_36_]^76^ [19], [Ce_20_Ge_10_W_100_O_376_(OH)_4_(H_2_O)_30_]^56−^ [20], [Gd_8_As_12_W_124_O_432_(H_2_O)_36_]^60−^ [15], [Gd_6_As_6_W_65_O_229_(OH)_4_(H_2_O)_12_(OAc)_2_]^38−^ [21], [Yb_10_As_10_W_88_O_308_(OH)_8_(H_2_O)_28_(OAc)_4_]^40−^ [21], [Na ⊂ Ln_12_Ge_6_W_60_O_228_(H_2_O)_24_]^35−^ (Na ⊂ Ln_12_) (Ln = Pr, Nd) [22], [K ⊂ Sm_12_Ge_6_W_60_O_228_(H_2_O)_22_]^35−^ [22], and [K ⊂ K_7_Ln_24_Ge_12_W_120_O_444_(OH)_12_(H_2_O)_64_]^52−^ (K ⊂ Ln_24_) (Ln = Pr, Nd) [22], which is due to the fact that lanthanide ions are large in size so cannot be fully incorporated into the lacunary sites of POMs as a result of their large coordination number, in turn leading to binding with terminal oxygen atoms of adjacent POM subunits and resulting in exceptionally giant and complex architectures. Heterometallic POMs (3d-4f-POM) is one of the least explored subclass of POMs, and so far, only a few POMs have been reported where 3d-4f-heterometallic clusters are stabilized by POM ligand [16,17]. On the other hand classical heterometallic coordination chemistry has been receiving a great deal of attention in the field of molecular magnetism, because of their potential advantages to create new single-molecule magnets. In such complexes, 3d ions can give rise to high-spin ground state (S), while 4f ions, such as Dy^III^, Tb^III^, Ho^III^, etc. import large anisotropic magnetic moments [23]. Remarkably recently we have successfully prepared a giant novel heterometallic polyanion {Dy_30_Co_8_Ge_12_W_108_O_408_} with single-molecule-magnet behavior. This polyanion contains the largest number of 4f ions besides being the first polyanion which interestingly incorporates two different type of metallic assemblies; six triangular {Dy_3_}, and four trigonal-bipyramidal {Co_2_Dy_3_}, within same POM framework [17]. Contrarily, often in the presence of 4f and 3d metal ions “4f-POM with 3d linker” and “3d-POM with 4f linker” type aggregates are formed due to the much higher reactivity and larger size of the oxophilic 4f ions compare to the 3d metal ion in the reaction mixture. For instance {[Cu(en)_2_]_2_(H_2_O)[Cu(en)(2,2′-bipy)]Ln[(*α*-HPW_11_O_39_)_2_]}^4−^ (2,20-bipy = 2,20-bipyridine and en = ethylenediamine) [24], [Cu(en)_2_]_5_[Cu(en)_2_(H_2_O)]_2_[RE_4_Ge_4_W_46_O_164_(H_2_O)_3_]^10−^ [25], and {[Cu(dap)_2_(H_2_O)]_2_[Cu(dap)_2_][*α*-XW_11_O_39_Ln(H_2_O)_3_]_2_}^4−^ (dap = 1,2-diaminopropane) [26], are some of the cases which come under the category of “4f-POM with 3d linkers” type POMs. Up to date to the best of our knowledge [(*γ*-SiW_10_O_36_)_2_(Cr(OH)(H_2_O))_3_(La(H_2_O)_7_)_2_]^4−^ [27], [{Ce(H_2_O)_7_}_2_Mn_4_Si_2_W_18_O_68_(H_2_O)_2_]^6−^ [28], [{Ln(H_2_O)_n_}_2_{Mn_4_(*B-α*-SiW_9_O_34_)_2_(H_2_O)_2_}]^6−^ (Ln = La^III^, Nd^III^, Gd^III^, Dy^III^, Er^III^) [29], and [Ce_4_(H_2_O)_22_(dpdo)_5_](Mn_2_HP_2_W_15_O_56_)_2_]^2−^ (dpdo = 4,4′-bipyridine-N,N′-dioxide) [30], are the only examples that fall under “3d-POM with 4f linkers” sub class.

The construction of POM-based materials is usually controlled by various factors, such as pH, reaction time, temperature, concentration, and ionic strength, but most important is the right selection of building blocks (POM precursor) and the right choice of metals centers to induce optical, electronic, and magnetic properties. One of the promising strategies for the production of all inorganic frameworks is to use 3d metal-substituted POMs with lanthanide metal centers, usually this combination has the potential to form one-, two- and three-dimensionally ordered structures. In this aspect, lanthanide cations are excellent candidates as linkers due to their high coordination numbers and Lewis acidity (*vide supra*). Additionally grafting of lanthanide cations also imparts interesting luminescent and magnetic properties to these hybrid nanostructures [31,32]. Despite rapid progress being made in the field of metal-organic frameworks (MOFs), POM-based frameworks have been receiving increasing attention, especially in the fields of selective adsorption, gas sensor, ion exchange, catalysis, optics, and magnetism [2,7,8]. Most importantly, all inorganic POM-frameworks promise to associate the thermal stability and general applicability of classical MOFs. POM-based porous inorganic frameworks would display higher thermal stability in comparison to classical MOFs because the thermal and chemical stability of the MOFs depend on the nature of the organic constituents/linkers, which might be destroyed during the activation of the porous material through elimination of solvent molecules in frameworks [33]. These difficulties could be overcome by the introduction of rigid metal clusters anions, such as POMs and lanthanide cations to the system. Moreover extensive- and additive properties of such materials can be finely tuned through variation of POM precursors and transition metal centers, respectively. 

Following this idea, we selected the trilacunary Wells–Dawson type POM ligand, Na_12_[*α*-P_2_W_15_O_56_]·24H_2_O (P_2_W_15_) [34], as a building block unit and introduced Dy^III^ ions and Mn^II^ ions cations to build up POM-based heterometallic extended frameworks.

Herein, we report a facile and convenient “one-pot” procedure for the synthesis of a heterometallic all inorganic system Na_10_(OH_2_)_42_[{Dy(H_2_O)_6_}_2_Mn_4_P_4_W_30_O_112_(H_2_O)_2_]·17H_2_O **(Dy_2_Mn_4_-P_2_W_15_**), which forms a framework structure, and so we designate as a Polyoxometalate Inorganic Framework, or POMIF. The title compound was structurally and compositionally characterized in solid state by single-crystal XRD, powder X-ray diffraction, Fourier-transform infrared spectroscopy, and thermal and elemental analyses. In solution state, UV–vis absorption spectroscopy and luminescence spectroscopy were performed. Furthermore, in order to check the bulk purity and to probe the solution state stability of the **Dy_2_Mn_4_-P_2_W_15_** comparative studies were performed with the well-known thermodynamically favorable and stable POM [Mn_4_(H_2_O)_2_(*α*-P_2_W_15_O_56_)_2_]^16−^ (**Mn_4_-P_2_W_15_**), a building block that is part of the [{Dy(H_2_O)_6_}_2_Mn_4_P_4_W_30_O_112_(H_2_O)_2_]^10−^. In addition, magnetic properties were studied and compared with that of Na_16_[Mn_4_(H_2_O)_2_(*α*-P_2_W_15_O_56_)_2_]·53H_2_O.

## 2. Experimental

The POM ligand, Na_12_[*α*-P_2_W_15_O_56_]·24H_2_O [34], and Na_16_[Mn_4_(H_2_O)_2_(*α*-P_2_W_15_O_56_)_2_]·53H_2_O [35], were synthesized according to the literature methods, and were characterized by IR spectroscopy. All reactions were carried out under aerobic conditions. All of the other reagents were purchased commercially and were used without further purification.

### 2.1. Synthesis

Synthesis procedure for **Dy_2_Mn_4_-P_2_W_15_**: MnCl_2_·4H_2_O (0.12 g, 0.6 mmol) was dissolved in 20 mL of water. Then Na_12_[*α*-P_2_W_15_O_56_]·24H_2_O (0.88 g, 0.1 mmol) was added to the above solution. Then, 0.30 mL of 1 M Dy(NO_3_)_3_·5H_2_O (0.13 g, 0.3 mmol) was added to this solution in small portions. The pH value of the mixture was adjusted to 7.6 by adding CH₃COONa (0.50 g, 6 mmol) in small portions under stirring. The resultant turbid orange solution was stirred at room temperature for 10 min, and then heated for 1 h at 80 °C. The resulting solution was filtered and left to slowly evaporate at room temperature, and orange crystals were obtained after approximately three weeks. Yield 229.8 mg (49% based on W). IR (2% KBr pellet, *ν*/cm^−1^): 1622 (m), 1082 (s), 1038 (w), 922 (s), 872 (w), 765 (w), 702 (w), 595 (w), 515 (m). Elemental analysis (%) calc. for Na_10_(OH_2_)_42_[{Dy(HO_2_)_6_}_2_Mn_4_P_4_W_30_O_112_(H_2_O)_2_]·17H_2_O (found): Na 2.34 (2.02), Mn 2.28 (2.56), W 57.17 (57.20), P 1.26 (1.28), Dy 3.37 (3.65).

### 2.2. Methods

Elemental analyses were performed at the Institute for Applied Materials, Karlsruhe Institute of Technology. Fourier transform IR spectra were measured on a Perkin-Elmer Spectrum One Spectrometer with samples prepared as KBr discs at the Institute of Nanotechnology, Karlsruhe Institute of Technology. X-ray powder diffraction patterns were measured at room temperature using a Stoe STADI-P diffractometer with a Cu-K*α* radiation at the Institute of Nanotechnology, Karlsruhe Institute of Technology. UV-vis spectra and fluorescent spectra were recorded on Cary 500 UV-vis-NIR spectrophotometer and Cary Eclipse fluorescence spectrophotometer, respectively, at the Institute of Nanotechnology, Karlsruhe Institute of Technology.

#### 2.2.1. Crystallography

Data were measured at 180(2) K on a Rigaku Oxford Diffraction SuperNova E diffractometer (Rigaku Europe, Kemsing, UK) with Mo-K*α* radiation from a microfocus source, and corrected semi-empirically for absorption. Structure solution was by dual-space direct-methods (SHELXT) [36], followed by full-matrix least-squares refinement (SHELX-2016) [37], with anisotropic thermal parameters for all the ordered non-H atoms. In a few cases, rigid-bond restraints (RIGU) were applied to the thermal parameters of mutually-bonded sodium cations and aquo oxygen atoms. Some of the lattice waters and aquo ligands coordinated to sodium cations were disordered; these were was modelled using pairs of isotropic partial-occupancy (0.60 and 0.40) oxygen atoms, with similarity restraints (SADI) applied to Na-O bond lengths where necessary. No attempt was made to locate any water H-atoms. Further details of the X-ray structural analysis are given in Table 1.

Further details of the crystal structure investigation may be obtained from FIZ Karlsruhe, 76344 Eggenstein-Leopoldshafen, Germany (Fax: (+49)7247-808-666; e-mail: crysdata@fiz-karlsruhe.de, on quoting the deposition number CSD-433863).

#### 2.2.2. Magnetic Measurements 

Magnetic susceptibility measurements were collected using a Quantum Design MPMS3 and MPMS-XL SQUID magnetometer (Quantum Design, San Diego, CA, USA). DC susceptibility measurements for all of the compounds were performed at temperatures ranging from 2 to 300 K, using an applied field of 1 kOe. Magnetization versus field data was collected between 2 and 5 K with applied field between 0 and 7 T. The AC data were collected using an oscillating magnetic field of 3.5 Oe. All data were corrected for diamagnetic contributions from the eicosane and core diamagnetism, estimated using Pascal’s constants [38].

## 3. Results and Discussion

### 3.1. Synthesis 

The heterometallic compound Na_10_(OH_2_)_42_[{Dy(H_2_O)_6_}_2_Mn_4_P_4_W_30_O_112_(H_2_O)_2_]·17H_2_O (**Dy_2_Mn_4_-P_2_W_15_**) was prepared by reaction of Mn^II^ ions and Dy^III^ ions with the trilacunary Wells-Dawson type [*α*-P_2_W_15_O_56_]^12−^ POM in aqueous basic media at 80 °C. It is important to note that the isolation of **Dy_2_Mn_4_-P_2_W_15_** was only possible in presence of excess of sodium acetate. The addition of Na_3_PO_4_, NaOH and Na_2_CO_3_ in the reaction mixture systems, while keeping the pH at 7.6, led to the formation of Na_16_[Mn_4_(H_2_O)_2_(*α*-P_2_W_15_O_56_)_2_]·53H_2_O [35]. In order to avoid one of the major difficulties, which is the formation of precipitate, due to the reaction of highly oxophilic lanthanides ions with highly negative and oxygen rich system like POMs, sodium acetate, and Dy^III^ ions were added slowly in portions. This illustrates the challenges of rationalizing the mechanistic pathways of heterometallic POM isolation in the form of crystals. To the best of our knowledge, **Dy_2_Mn_4_-P_2_W_15_** not only represents the first all inorganic heterometallic 3D POMIF compound based on Wells-Dawson type [*α*-P_2_W_15_O_56_]^12−^, but also provides a feasible route to the formation of highly robust multifunctional extended structures base on POMs.

### 3.2. Structure

**Figure 1:**
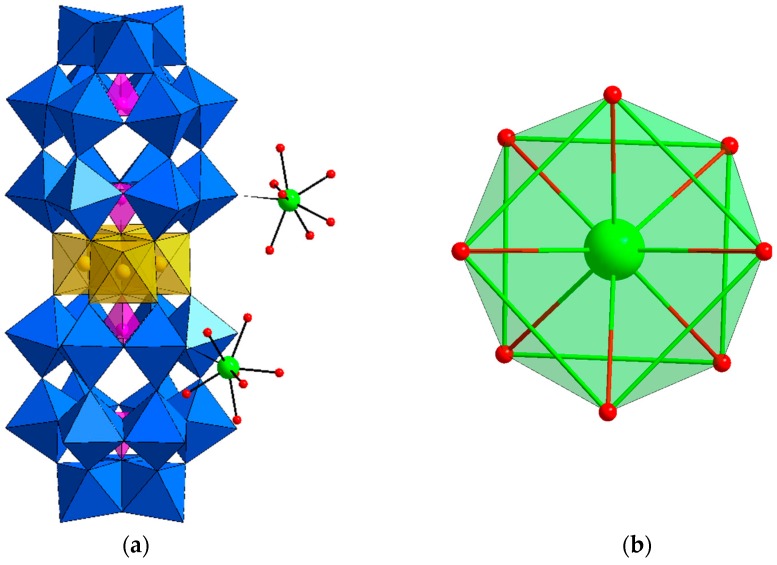
(**a**) Polyhedral and ball-and-stick representation of the sandwich-type polyanion and the dysprosium cations of **Dy_2_Mn_4_-P_2_W_15_**; (**b**) Local geometry around the dysprosium cation in **Dy_2_Mn_4_-P_2_W_15_**, possessing a square antiprism geometry. Color scheme: Dy = green; O = red; WO_6_ octahedron = blue, MnO_6_ octahedron = gold; PO_4_ tetrahedron = Pink.

**Figure 2:**
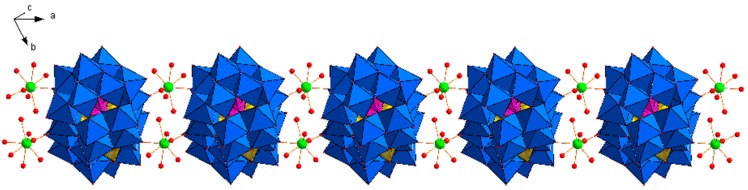
View of the one-dimensional (1D) ladder like chain in **Dy_2_Mn_4_-P_2_W_15_**. Color scheme: Dy = green; O = red; WO_6_ octahedron = blue, MnO_6_ octahedron = gold; PO_4_ tetrahedron = Pink.

**Figure 3:**
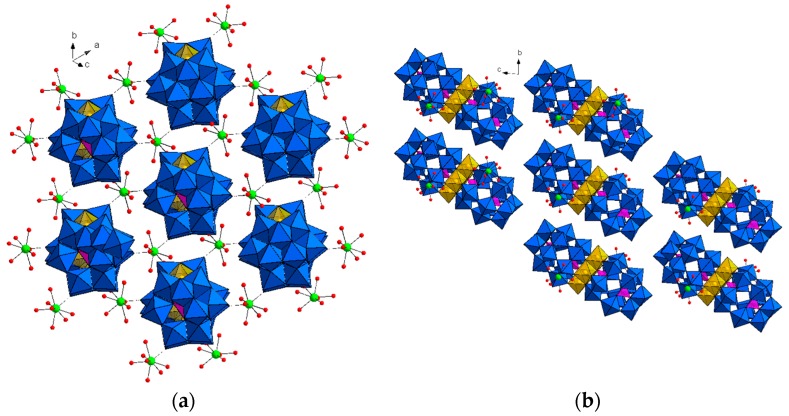
(**a**) The two-dimensional (2D) network packing arrangement in **Dy_2_Mn_4_-P_2_W_15_**; (**b**) packing of **Dy_2_Mn_4_-P_2_W_15_** viewed down the a-axis. Crystal water molecules and sodium cations are omitted for clarity. Color scheme: Dy = green; O = red; WO_6_ octahedron = blue, MnO_6_ octahedron = gold; PO_4_ tetrahedron = Pink.

Single-crystal X-ray diffraction revealed that **Dy_2_Mn_4_-P_2_W_15_** crystallizes in the triclinic system with space group P$\bar{1}$, and consists of tetranuclear manganese-substituted sandwich-type [Mn_4_(H_2_O)_2_(*α*-P_2_W_15_O_56_)_2_]^16−^ (**Mn_4_-P_2_W_15_**), Na and Dy^III^ cations (Figure 1). The crystal structure can be viewed as a one-dimensional (1D) ladder-like chain, built up of the sandwich anions **Mn_4_-P_2_W_15_** and the Dy^III^ ions (Figure 2). Each Dy^III^ center connects two **Mn_4_-P_2_W_15_** subunits via two W═O···Dy bonds and the bond lengths are in the range 2.31–2.42 Å. The Dy^III^ ions are octacoordinate and possess square antiprism geometry (Figure 1). The coordination environment is completed by six terminal water molecules and two bridged oxygen atoms from two sandwich-type polyoxoanion. Furthermore, the Dy^III^ ions can be viewed as *μ*_2_-bridges which link two **Mn_4_-P_2_W_15_** into a 1D network structure which are further extended into a three-dimensional open framework by the sodium cations (Na_1_, Na_4_, and Na_5_), while Na_2_ and Na_3_ are just charge-balancing segments on the surface of the POM. The adjacent ladder-like chains are connected by the sodium cations to form a two-dimensional framework (Figure 3). Until now, there are only few reports on the heterometallic extended aggregates systems where 3d metal substituted sandwich-type POMs are linked by lanthanide cations, for instance [(*γ*-SiW_10_O_36_)_2_(Cr(OH)(H_2_O))_3_(La(H_2_O)_7_)_2_]^4−^ [27], [{Ce(H_2_O)_7_}_2_Mn_4_Si_2_W_18_O_68_(H_2_O)_2_]^6−^ [28], [{Ln(H_2_O)_n_}_2_{Mn_4_(*B-α*-SiW_9_O_34_)_2_(H_2_O)_2_}]^6−^ (Ln = La, Nd, Gd, Dy, Er) [29] and [Ce_4_(H_2_O)_22_(dpdo)_5_](Mn_2_HP_2_W_15_O_56_)_2_]^2−^ (dpdo = 4,4′-bipyridine-N,N′-dioxide) [30]. So far to the best of our knowledge, the only heterometallic 3D POMOF based on trilacunary Wells-Dawson type POM is [Ce_4_(H_2_O)_22_(dpdo)_5_](Mn_2_HP_2_W_15_O_56_)_2_]^2−^ (dpdo = 4,4′-bipyridine-N,N′-dioxide), which is composed of sandwich type precursor with formula [Mn_4_(HP_2_W_15_O_56_)_2_]^14−^, two Na ions, two Ce^III^ cations, and three dpdo ligands [30]. Except [{Ln(H_2_O)n}_2_{Mn_4_(*B-α*-SiW_9_O_34_)_2_(H_2_O)_2_}]^6−^, in all of the above reported aggregates, transition metal substituted sandwich-type POMs were used as precursor, while in our case, **Mn_4_-P_2_W_15_** can be formed in situ. The sandwich-type building block, **Mn_4_-P_2_W_15_** is constructed by two trivacant Dawson moieties [*α*-P_2_W_15_O_56_]^12−^ sandwiching a regular rhomb-like cluster {Mn_4_O_16_(H_2_O)_2_} through the W–O–Mn and P–O–Mn bridging modes. All of the W and Mn^II^ centers display an octahedral coordination environment. The bond lengths of W–O are in the range 1.71–2.39 Å, whereas the bond lengths of Mn–O are between 2.09 Å and 2.29 Å. 

### 3.3. Characterizations

**Figure 4:**
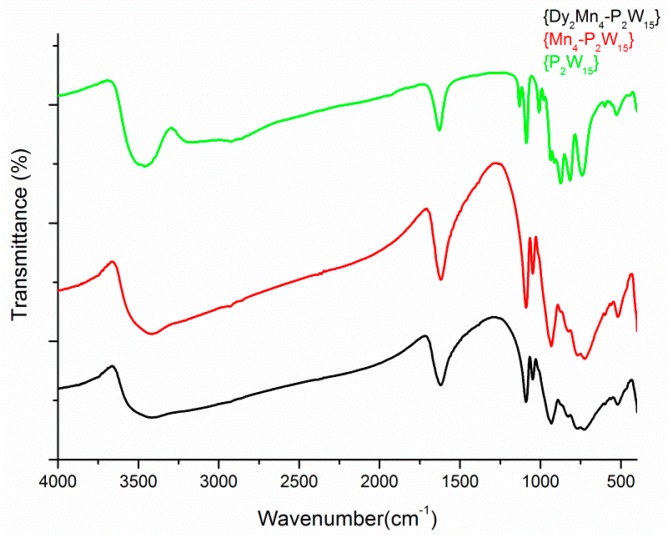
Fourier transform infrared (FT-IR) spectroscopy of **Dy_2_Mn_4_-P_2_W_15_**, **Mn_4_-P_2_W_15_**, and **P_2_W_15_**.

Infrared is spectroscopy is one of the most frequently employed techniques for the characterization of polyoxometalates, due to its characteristic peaks in the in the region (1200–450 cm^−1^), which is called the fingerprint region for the POM backbone [39]. The similarity in the IR spectra of Na_10_(OH_2_)_42_[{Dy(H_2_O)_6_}_2_Mn_4_P_4_W_30_O_112_(H_2_O)_2_]·17H_2_O and Na_16_[Mn_4_(H_2_O)_2_(*α*-P_2_W_15_O_56_)_2_]·53H_2_O (Figure 4) suggests that the attachment of the Dy^III^ cations on the **Mn_4_-P_2_W_15_** surface has not changed the vibration modes in **Dy_2_Mn_4_-P_2_W_15_**, except for some minor shifts that are observed due to the influence of the dysprosium linkers (Figure 4).

**Figure 5:**
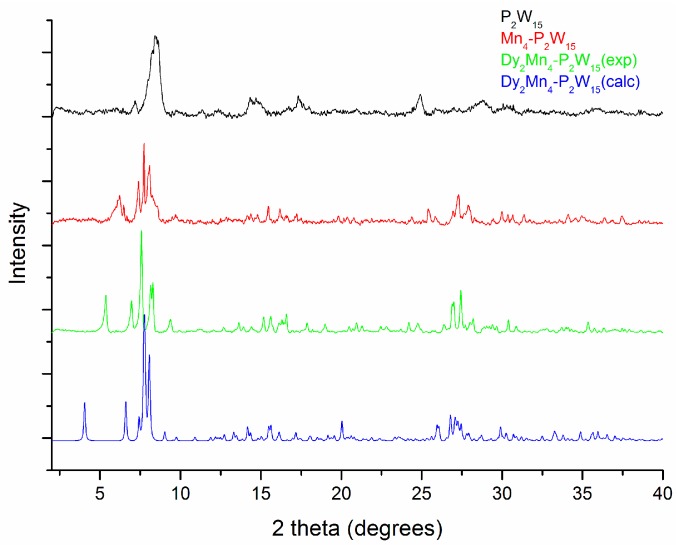
Stimulated and experimental powder X-ray diffraction (PXRD) patterns of **Dy_2_Mn_4_-P_2_W_15_**, along with experimental PXRD patterns of **Mn_4_-P_2_W_15_**, and **P_2_W_15_**.

Since the FTIR studies were not a helpful tool to check the bulk purity of the product in this case, we decided to carry out the powder X-ray diffraction (PXRD) analysis for the compounds **Dy_2_Mn_4_-P_2_W_15_**, **Mn_4_-P_2_W_15_** and **P_2_W_15_**, in order to examine the bulk purity of the title product (Figure 5). The experimental PXRD pattern of **Dy_2_Mn_4_-P_2_W_15_** corresponds well to the simulated PXRD pattern, indicating that the bulk phase materials are isomorphous. The very minor differences in peak intensity and 2 theta values are likely due to the loss of some lattice water molecules, bearing in mind that the simulated patterns are generated from the single crystal X-ray diffraction data set, which was collected at 100 K.

**Figure 6:**
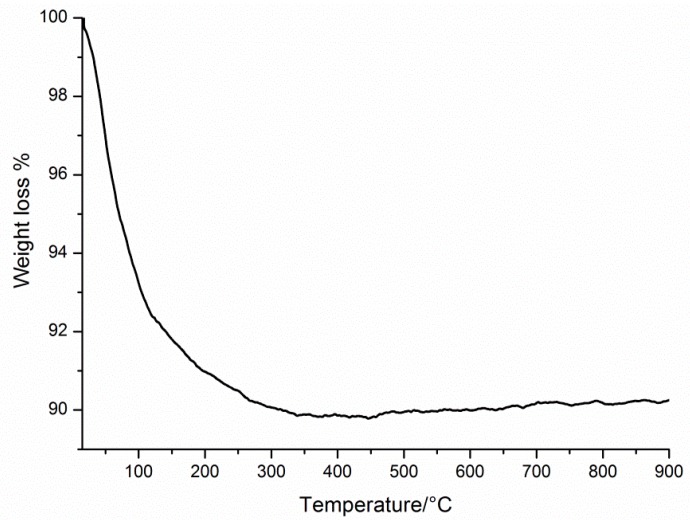
Thermogram of **Dy_2_Mn_4_-P_2_W_15_** from room temperature to 900 °C.

Thermogravimetric analysis of **Dy_2_Mn_4_-P_2_W_15_** was performed between 20 and 900 °C under a nitrogen atmosphere to determine the number of crystal waters (Figure 6). The continuous weight loss of about 10% between 25 and 330 °C can be attributed to the loss of all lattice and coordinated water molecules present in **Dy_2_Mn_4_-P_2_W_15_**.

Elemental analysis on Mn, Dy, O, W, P, and Na was also carried out by means of inductively coupled plasma optical emission spectrometry, and the results are presented in Section 2.1.

**Figure 7:**
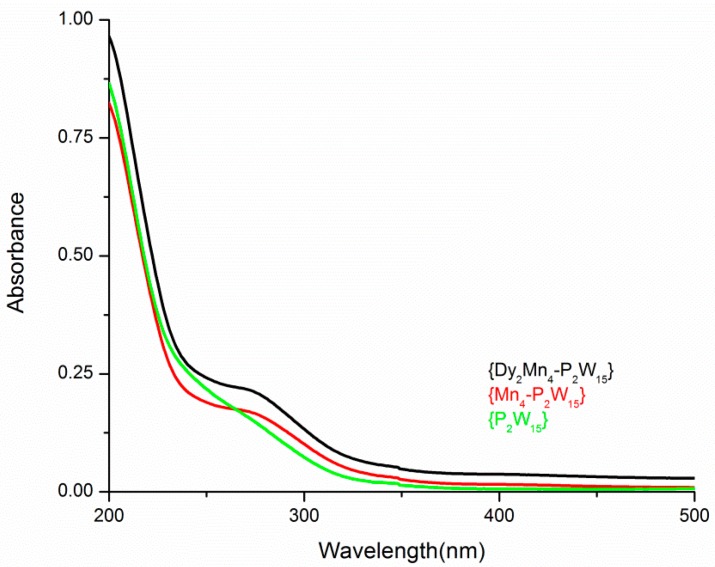
UV-vis spectra **Dy_2_Mn_4_-P_2_W_15_**, **Mn_4_-P_2_W_15_**, and **P_2_W_15_**.

Solution state UV−vis measurements were performed on **Mn_4_-P_2_W_15_**, **Dy_2_Mn_4_-P_2_W_15_** and **P_2_W_15_** in order to investigate the incorporation of metal centers in the polytungstate framework and to check the structural integrity in solution. The UV-Vis absorption spectra show that the **Dy_2_Mn_4_-P_2_W_15_** is like that of **Mn_4_-P_2_W_15_** in solution, which is one of the most stable POMs in solution. However the absorption spectra of **Dy_2_Mn_4_-P_2_W_15_** and **Mn_4_-P_2_W_15_** are significantly different from that of the **P_2_W_15_** precursor, which gives a clear indication that the incorporation of metal centers in the polytungstate framework has an extensive effect on the physicochemical properties of the resulting clusters. However, information about the possibility that there are coordinated Dy^III^ cations on the POM surface in solution phase cannot be achieved from these studies. The UV-Vis spectra (Figure 7) show absorbance bands between 200 and 400 nm, which can be associated with the characteristic charge-transfer bands of terminal oxygen and bridging oxygen atoms to tungsten centers, respectively (oxygen-to-metal charge transfer (O → M LMCT).

**Figure 8:**
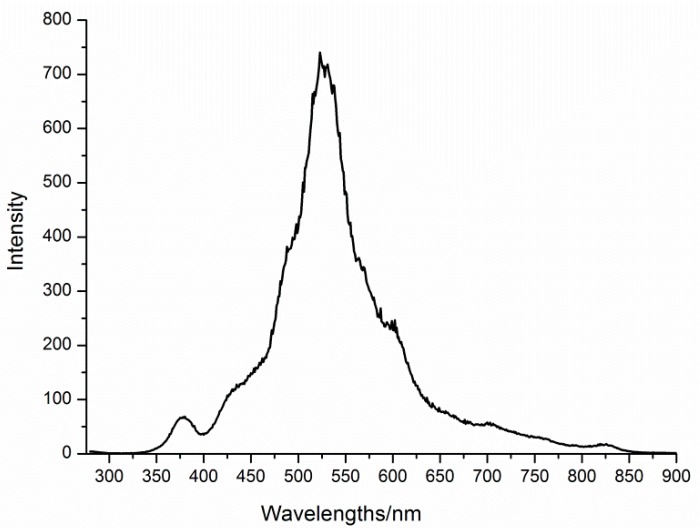
Emission spectrum (*λ*_ex_ = 275 nm) of **Dy_2_Mn_4_-P_2_W_15_** showing Dy^III^ emission.

**Figure 9:**
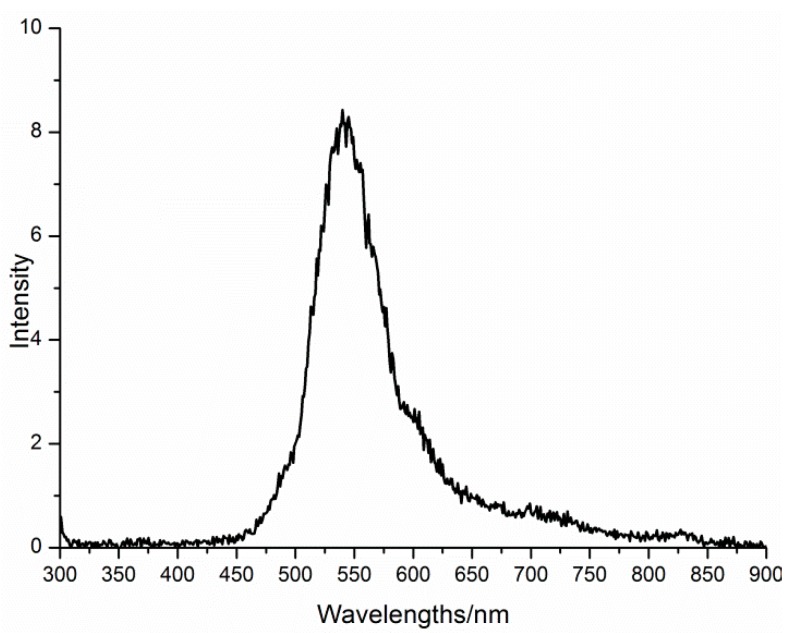
Emission spectrum (*λ*_ex_ = 275 nm) of **Mn_4_-P_2_W_15_**.

When compared to absorption studies, emission spectroscopy is an advantageous tool, particularly in any heterometallic system, to investigate the origin of the emission bands, since in comparison with the non-Ln containing materials, the Ln-containing systems often have higher luminescence efficiency, better photochemical stability, and prolonged fluorescence life times. The lanthanide’s emission is substantially induced by the photoexcitation of the oxygen-to-metal charge transfer (O → M LMCT) bands of 4f-POM systems [31,40]. Consequently, in order to gain insight on the solution behavior of the title compound, the luminescent properties of **Dy_2_Mn_4_-P_2_W_15_** and **Mn_4_-P_2_W_15_** were measured at room temperature under the same excitation of 275 nm. As shown in Figure 8, **Dy_2_Mn_4_-P_2_W_15_** displays an intense characteristic photoluminescence emission band (450–650 nm wide) centered at 545 nm, which can be assigned to the ^4^F_9/2_ → ^6^H_13/2_ and ^4^F_9/2_ → ^6^H_11/2_ transitions in Dy^III^ ions, while the weak emission of Dy^III^ ions at 379 can be attributed to the ^4^F_9/2_ → ^6^H_15/2_ transition [41]. It should also be noted that the Dy^III^ emission bands (450–650 nm) overlap with the characteristic broad Mn^II^ emission band (Figure 9) (ca. 500–600 nm) [42], and therefore are not distinguishable. The presence of the emission band of Dy^III^ ions at 379 nm (only observed in the photoluminescence spectrum of **Dy_2_Mn_4_-P_2_W_15_**) (Figure 8), indicating the existence of [{Dy(OH_2_)_6_}_2_Mn_4_P_4_W_30_O_112_(H_2_O)_2_]^10−^ in solution. However, whether the extended framework is preserve in solution cannot be determined from these data. Further, it can also be observed that the emission profile slightly changes and emission intensity increases drastically in the case of **Dy_2_Mn_4_-P_2_W_15_**. Although the solutions were only weakly luminescent, distinct differences could be observed not only in the emission intensity, but also in the number of emissions bands. 

## 4. Magnetic Properties

**Figure 10:**
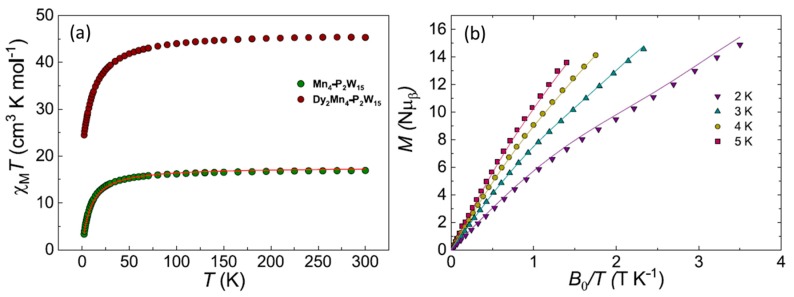
(**a**) Experimental *χ*_M_*T* v T curves for **Mn_4_-P_2_W_15_** and **Dy_2_Mn_4_-P_2_W_15_**, with best fit (red line) for **Mn_4_-P_2_W_15_**; (**b**) The reduced magnetization and best fits (solid lines) at given temperatures employing parameters in the text.

To further characterize the physical properties of both POM complexes, SQUID measurements were conducted on polycrystalline materials. Firstly, we studied the static magnetic behavior of the building block POM unit using neat polycrystalline powders under an applied dc field of 1 kOe in the temperature range 2 to 300 K (Figure 10a). The room temperature *χ*_M_*T* product (where *χ*_M_ is molar magnetic susceptibility) for the building block is close to the expected value for four isolated Mn^II^ ions for **Mn_4_-P_2_W_15_**, i.e., 16.8 cm^3^ K mol^−1^ (cf. 17.5 cm^3^ K mol^−1^ for four Mn^II^, *s* = 5/2, *g* = 2.00). Upon cooling, the *χ*_M_*T* product stays constant down to ca. 50 K, after which it smoothly decreases reaching 3.2 cm^3^ K mol^−1^ at 1.8 K, indicating that antiferromagnetic interactions are be operative within the cluster. Furthermore, we studied the molar magnetization (*M_β_*) as function of applied magnetic field at 2 and 5 K in the field range 0–7 T. The *M_β_* versus applied field, H, for the compound **Mn_4_-P_2_W_15_,** indicates a strong antiferromagnetic interaction, as observed in the almost linear behavior of the magnetization curves. The *M_β_*(*H*) data at the maximum field (7 T) and the lowest temperature (2 K) yield a value of 14.8 *μ_β_* (Figure 10b). The isotropic nature of the compound **Mn_4_-P_2_W_15_** allows for us to simultaneously fit the *χ*_M_*T*(*T*) and *M_β_*(*H*) using the simple Heisenberg Hamiltonian, taking into account two exchange interactions, i.e., $\hat{H}=\;-2J_{1}{\hat{S}}_{1}∙{\hat{S}}_{2}-2J_{2}\left({\hat{S}}_{1}∙{\hat{S}}_{3}+{\hat{S}}_{1}∙{\hat{S}}_{4}+{\hat{S}}_{2}∙{\hat{S}}_{3}+{\hat{S}}_{2}∙{\hat{S}}_{4}\right)+g\mu_{B}H\sum_{i=1}^{4}{\hat{S}}_{i}$. A single *g*-value 2.025 for all Mn^II^ ions and two small interactions of similar magnitude reproduce the small down turn in *χ*_M_*T*(*T*) and *M_β_*(*H*) (solid lines in Figure 10), i.e., *J*_1_ = −0.463(1) cm^−1^ between nearest Mn^II^ ions, while *J*_2_ = −0.342(1) cm^−1^ for the Mn^II^ at longer distances. These values are in line with those found in previous work [35].

**Figure 11:**
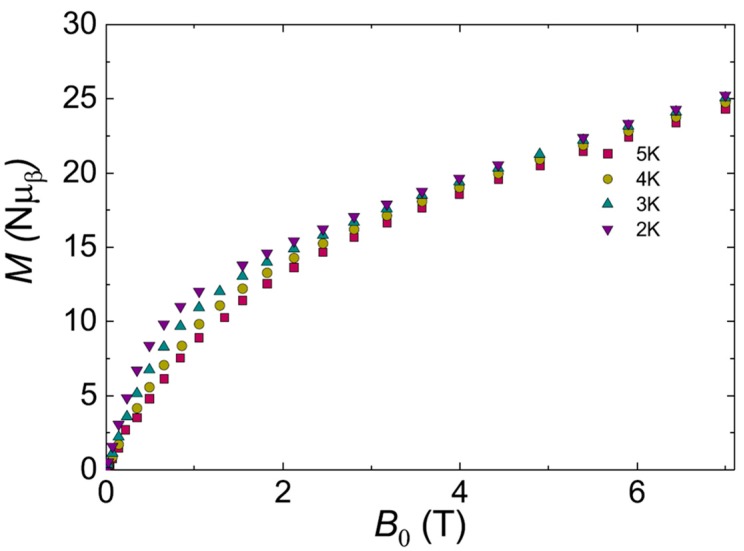
Experimental magnetization versus field at different temperatures (*M*(*H*,*T*)) for **Dy_2_Mn_4_-P_2_W_15_**.

After magnetically characterizing the building block POM, we proceed with the study of the magnetic properties of the POMIF, i.e., **Dy_2_Mn_4_-P_2_W_15_**. As can be observed in Figure 10a, the *χ*_M_*T*(*T*) shows a room temperature *χ*_M_*T* value of 45.3 cm^3^ K mol^−1^ close to the value that is expected for the building block and two non-interacting dysprosium ions, i.e., 45.8 cm^3^ K mol^−1^ for four Mn(II), *s* = 5/2, g = 2.00 and two Dy^III^, *J* = 15/2; *g_J_* = 4/3). Likewise, the *χ*_M_*T* product starts decreasing below 100 K, reaching a minimum value of 24.5 cm^3^ K mol^−1^. In this case, the drop observed in the *χ*_M_*T* product could arise from a combined effect of the antiferromagnetic exchange operating in the **Dy_2_Mn_4_-P_2_W_15_** unit along with the anisotropic magnetic properties of Dy^III^ ions, that is, depopulation of the ligand field levels. The *M_β_*(*H*) for compound **Dy_2_Mn_4_-P_2_W_15_** similarly shows very anisotropic behavior and no saturation of the *M_β_*(*H*) (see Figure 11), with a *M_β_* value of 25.3 *μ_β_* at the maximum field (7 T) and the lowest temperature (2 K). Additionally, we have tested the dynamic behavior of this complex at 2 K in the frequency range 1–1500 Hz without and with applied dc fields (ranging from 0 to 5 kOe), and an oscillating magnetic field of 3.5 Oe. No out of phase component in could be observed within the measurement parameters of our equipment and there is SMM behavior under these conditions.

## 5. Conclusions

In conclusion, an all inorganic heterometallic extended framework incorporating POM units has been synthesized in aqueous media under “one-pot” conditions. This Polyoxometalate Inorganic Framework, or POMIF, [{Dy(HO_2_)_6_}_2_Mn_4_P_4_W_30_O_112_(H_2_O)_2_]^10−^ (**Dy_2_Mn_4_-P_2_W_15_**), was prepared by reaction of Mn^II^ ions and Dy^III^ ions with the trilacunary Dawson ion [*α*-P_2_W_15_O_56_]^12−^. The hydrated salt of **Dy_2_Mn_4_-P_2_W_15_** was structurally characterized in the solid state by single-crystal X-ray diffraction, X-ray powder diffraction (XRD), Fourier transform infrared (FTIR) spectroscopy, and thermal and elemental analyses and in solution by UV-Visible absorption spectroscopy and luminescence emission spectroscopy. Furthermore, magnetic properties were also studied and compared with Na_16_[Mn_4_(H_2_O)_2_(*α*-P_2_W_1S_O_56_)_2_]·53H_2_O. We are presently attempting to synthesize analogs containing various combinations of 3d and 4f elements in order to do systematic and comparative studies of the resultant frameworks.

## Figures and Tables

**Table 1:** Crystal Data.

Compound	Dy_2_Mn_4_-P_2_W_15_
Formula	Dy_2_H_146_Mn_4_Na_10_O_185_P_4_W_30_
Formula weight	9521.20
Crystal System	Triclinic
Space Group	P$\bar{1}$
*a*/Å	13.3544(4)
*b*/Å	14.7907(4)
*c*/Å	22.5101(6)
*α*/°	79.938(2)
*β*/°	76.843(2)
*γ*/°	65.236(3)
V/Å^3^	3915.5(2)
*Z*	2
T/K	180(2)
Crystal dimensions/mm	0.11 × 0.06 × 0.02
*F*(000)	4248
*D_c_*/Mg m^−3^	4.038
*μ*(Mo-K*α*)/mm^−1^	23.389
Data Measured	49,270
Unique Data	17,079
*R_int_*	0.0427
Data with I ≥ 2*σ*(I)	14,193
*wR*_2_ (all data)	0.2055
*S* (all data)	1.083
*R*_1_ [I ≥ 2*σ*(I)]	0.0732
Parameters/Restraints	1045/29
Biggest diff. peak/hole/eÅ^−3^	+4.53/−1.87
FIZ-CSD number	433863
